# Adhesion of Polypropylene, Steel, and Basalt Fibres to a Geopolymer Matrix with Water Treatment Sludge Addition

**DOI:** 10.3390/ma18204727

**Published:** 2025-10-15

**Authors:** Mateusz Sitarz, Tomasz Zdeb, Tomasz Tracz, Michał Łach

**Affiliations:** 1 Chair of Building Materials, Faculty of Civil Engineering, Cracow University of Technology, ul. Warszawska 24, 31-155 Kraków, Poland; tomasz.zdeb@pk.edu.pl (T.Z.); tomasz.tracz@pk.edu.pl (T.T.); 2 Interdisciplinary Center for Circular Economy, Cracow University of Technology, ul. Warszawska 24, 31-155 Kraków, Poland; michal.lach@pk.edu.pl; 3 Department of Materials Engineering, Faculty of Materials Science and Physics, Cracow University of Technology, al. Jana Pawła II 37, 31-864 Kraków, Poland

**Keywords:** alkali-activated materials, geopolymers, fibres, adhesion, sustainability

## Abstract

This study investigates the adhesion of polypropylene (PP), steel and basalt fibres to geopolymer matrices of varying composition. Geopolymers formed via alkali activation of fly ash (FA) and ground granulated blast-furnace slag (GGBFS) offer significant environmental advantages over Portland cement by reducing CO_2_ emissions and energy consumption. The addition of water treatment sludge (WTS) was also investigated as a partial or complete replacement for FA. Pull-out tests showed that replacing FA with WTS significantly reduces the mechanical properties of the matrix and at the same time the adhesion to the fibres tested. The addition of 20% WTS reduced the compressive strength by more than 50% and full replacement to less than 5% of the reference value. Steel fibres showed the highest adhesion (9.3 MPa), while PP fibres had the lowest, with adhesion values three times lower than steel. Increased GGBFS content improved fibre adhesion, while the addition of WTS weakened it. Calculated critical fibre lengths ranged from 50 to 70 mm in WTS-free matrices but increased significantly in WTS-containing matrices due to reduced matrix strength. The compatibility of the fibres with the geopolymer matrix was also confirmed via SEM microstructural observations, where a homogeneous transition zone was observed in the case of steel fibres, while numerous discontinuities at the interface were observed in the case of other fibres, the surface of which is made of organic polymers. These results highlight the potential of fibre-reinforced geopolymer composites for sustainable construction.

## 1. Introduction

In the context of global sustainable construction, there is a growing focus on building materials that are clean, efficient, and durable. The annual production of Portland cement (OPC) is at least 4 billion metric tonnes [1]. It is estimated that cement production could reach 6 billion tonnes by 2050 [2]. Unfortunately, each tonne of OPC is associated with the emission of close to one tonne of CO_2_ into the atmosphere and the consumption of 10–11 EJ energy per year, which is about 3% of global energy consumption [3]. At the same time, about 5–7% of global carbon dioxide emissions come from the cement industry [4]. Moreover, OPC exhibits limited resistance to aggressive environmental conditions, which adversely impacts the durability and long-term performance of concrete structures. To reduce greenhouse gas emissions and energy consumption, it is essential to develop a high-performance, eco-friendly alternative binder.

The development of modern binders should go beyond improving mechanical performance. It should be directly linked to sustainable development goals, including the extension of composite durability and the use of waste raw materials. Recent studies on the incorporation of recycled carbon fibres into mineral mortars confirm the potential to enhance mechanical resistance while simultaneously reducing costs and environmental impact [5]. One example of this approach is the stabilization of sewage sludge with low-emission binders supplemented by industrial by-products such as FA and slags. This method not only enhances the load-bearing capacity and impermeability of the material but also substantially reduces CO_2_ emissions, while facilitating the utilization of environmentally problematic waste [6]. Moreover, replacing conventional cement additives with alternative materials derived from waste can effectively enhance the durability of composites under extreme conditions, while simultaneously reducing the consumption of natural resources [7]. Current research indicates that the development of modern binders should focus on achieving synergy between mechanical durability and the minimization of environmental impact.

As a result, significant research efforts have been directed toward the development of novel binders capable of replacing OPC. These efforts have led to the emergence of a new class of materials known as alkali-activated materials (AAMs), or geopolymers. Geopolymers are inorganic polymers typically formed by alkaline activation of aluminosilicate precursors [8]. Compared to the production of OPC, the production of geopolymers can produce 80% less carbon dioxide and require 60% less energy [9]. This large reduction is achieved by using waste and by-products from industry to manufacture geopolymer composites. Utilizing industrial by-products such as FA and slags in the synthesis of geopolymers contributes significantly to the environmental sustainability of binder systems. Moreover, numerous studies have been dedicated to investigating the properties and performance of geopolymer composites (GPCs). The results show excellent properties, such as high early strength [10], chemical resistance [11], excellent surface hardness [12], and high temperature and fire resistance [13,14]. 

Recent studies emphasize the crucial role of accurately selecting the initial parameters of raw materials in geopolymer production. The molar ratios of key components, such as CaO/Al_2_O_3_ and SiO_2_/Al_2_O_3_, are decisive in shaping the mechanical properties of geopolymer composites, particularly when incorporating industrial by-products. Appropriate adjustment of these parameters not only optimizes material strength but also reduces the carbon footprint, thereby fostering the development of sustainable technologies [15]. Increasing demands for durability and sustainability are driving the search for innovative construction materials. The latest research indicates that hybrid composites and advanced modeling techniques represent a key direction in the development of modern materials engineering [16,17].

As with cementitious materials, the mineral geopolymer matrix is relatively brittle and can be prone to cracking under tensile stress [18]. Therefore, improving the mechanical properties, such as toughness, tensile strength, and flexural strength, of geopolymer composites (GPCs) can significantly increase their use in the construction sector. An effective way to improve these parameters is to introduce fibres as reinforcement into the geopolymer matrix [19]. Fibre reinforcement may be applied as continuous strands or short fibres. In civil engineering, short fibres are preferred due to their economic advantages, ease of application, and compatibility with conventional mixing techniques. 

Numerous studies have investigated the incorporation of various fibre types into geopolymers. This reinforcement is characterized by the diversity of fibre materials (such as steel, synthetic, inorganic, and natural fibres) as well as by parameters including fibre content, aspect ratio (length-to-diameter), geometry, and physical and mechanical properties [20]. The incorporation of fibres significantly contributes to fracture control by mitigating the inherent brittleness of geopolymers and improving their post-cracking load-bearing capacity. Reinforcing fibres function as crack-bridging agents, arresting the growth of micro-cracks induced by tensile stresses [21,22]. Debonding, sliding, and pulling of fibres absorb a large amount of energy that could be used to create cracks. As a result, once the maximum strength is reached, the composite shows a significant increase in resistance due to the presence of fibres [23].

Research papers on fibre-reinforced geopolymers most often describe the use of steel or synthetic fibres. Among steel fibres, the most common fibres are those containing hooked-end and micro steel fibres. The steel used for fibre production is characterized by high tensile strength and a high elastic modulus. In addition, fibres containing hooked-end fibres provide additional mechanical anchorage during the debonding process [24]. In the case of synthetic fibres, the most commonly described fibres are polyvinyl alcohol (PVA), which are especially known for their high tensile strength, good elastic modulus and resistance to acids and alkalis. PP fibres are also widely used, primarily because of their affordability and ease of use [25,26]. Inorganic fibres, such as glass and basalt, are among the most frequently investigated due to their promising structural properties [27,28].

Another category comprises natural fibres, primarily plant-based, such as sisal, coir, cotton, hemp, flax, and jute. Currently, these fibres represent a minor share of the materials used in geopolymer reinforcement. Due to the growing interest in sustainability and environmentally friendly materials, composites with natural fibres have an opportunity for development [29,30]. 

The fibre–matrix interface plays a critical role in determining the mechanical performance of fibre-reinforced composites. The interfacial transition zone (ITZ) between the fibre and the surrounding matrix provides resistance through mechanisms such as chemical bonding and adhesion. During pull-out, the chemical bonding may provide the first mechanism to prevent the fibre from sliding out. In this initial phase of deformation, the fibre and matrix cooperate fully. The deformations in both phases are compatible, which prevents damage to the interface between them [31]. The properties of ITZ depend on the ratio of solution to binder, the type of binder used, and the additives or inclusions. The quality and bonding characteristics of the fibre–matrix interface are critical to the overall behaviour of the composite. In fibre-reinforced geopolymer concrete, the ITZ has a more pronounced influence on mechanical properties compared to unreinforced geopolymer concrete. This is primarily due to the significantly higher aspect ratio of fibres relative to aggregate particles, which results in a larger interfacial contact area.

The inclusion of GGBFS in geopolymer composites has been shown to enhance their mechanical and durability properties relative to systems based solely on FA [32]. The partial replacement of FA by GGBFS results in a qualitative change in the bonding reaction products. Geopolymerisation products that are strongly bonded to the fibre surfaces can increase the friction between the fibres and the matrix during fibre pull-out. This enhanced interfacial friction contributes to improved composite strength [9,33,34]. The chemical adhesion and bond strengths between the steel fibres and the geopolymer matrix observed during fibre pull-out are higher for geopolymers containing GGBFS [35]. Higher adhesion of steel fibres to the geopolymer matrix containing GGBFS, compared to cementitious matrices, was observed by researchers during strength testing [36,37]. This was also confirmed by observations of the interfacial zone between the fibre and the matrix. Based on SEM observations, a high homogeneity of the material structure around the steel fibres was observed. In addition, Bhutta et al. [38] showed that higher slag content in the geopolymer matrix, causes increased strength of the fibre–matrix interfacial zone.

In mineral composites, one of the key factors influencing the quality of the fibre–matrix bond is the wettability of the fibres with water [39]. Ranjbar et al. [40] conducted a study to compare the wettability of MS (micro steel) and PP fibres by determining the wetting angle of the fibres. According to the classification adopted, fibre surfaces are hydrophilic when the wetting angle is less than 90° and hydrophobic when this angle is greater than 90°. A lower wetting angle determines better adhesion of the fibres to the matrix. The results obtained by the authors show that, in the case of PP fibres, the initial contact angle after 30 s of water exposure is 98°. It then gradually decreases, reaching a value of 91.1° after 240 s of contact. The measured values (above 90 degrees) indicate the hydrophobic nature of PP fibres, which may explain the weaker bonding of PP fibres to the geopolymer matrix. Lower values were obtained for MS fibres. The wetting angle after 30 s was 85.5°, and after 240 s, the value dropped even further, reaching 69.3°. The results indicate that the fibre surface can be classified as hydrophilic, which contributes to a stronger bond between the fibre and the matrix. 

Among synthetic fibres, PP and polyvinyl alcohol (PVA) fibres are frequently considered for use in geopolymer composites [41,42]. In most cases, only a slight improvement in the performance of geopolymer composites reinforced with PP fibres is confirmed [43]. This is mainly due to the smooth surface of the PP fibres, which limits the bond strength at the interface between the fibres and the matrix. Furthermore, Masi et al. [44] showed better adhesion of synthetic PVA fibres compared to basalt fibres based on SEM observations. The PVA fibres were completely covered by the geopolymer gel and the longitudinal striations on their surface enhanced the bond between the fibre and the geopolymer matrix. In contrast, the smooth surface of basalt fibres resulted in a weak bond with the matrix, as evidenced by the absence of geopolymer products on the surface of the pulled-out fibres. Conversely, when PVA fibres were incorporated into the geopolymer matrix, their ends were largely covered with geopolymer products. This suggests a failure mechanism dominated by fibre pull-out, contributing to a more ductile failure mode. In comparison, the sharp cross-sections observed at the ends of basalt fibres indicate brittle failure [44].

The presented literature review shows the wide range of geopolymer composite properties that can be improved by the use of fibres. Fibre-reinforced geopolymers are able to behave more effectively during tensile (bending) loading. The presence of fibres significantly modifies the failure mode of the composite, shifting it from a brittle to a pseudo-ductile or pseudo-plastic response. 

The research part of this paper analyses the interaction of three types of fibres (steel fibres—anchored fibre, synthetic fibres—PP fibre, and mineral fibres—basalt fibre) with the different geopolymer matrices. The main goal of this paper was to determine the adhesion between fibres and matrices and, as a result, the critical length of individual fibres to optimise the design of the geopolymer fibre composite. Moreover, the performed pull-out studies were supplemented by SEM observations in the interfacial transition zone.

## 2. Materials and Methods

### 2.1. Fly Ash and Slag

The oxide composition of the raw materials was determined by XRF using an EDX-7200 spectrometer (Shimadzu Corp., Kyoto, Japan). XRD analysis was performed using an Aeris diffractometer (Malvern PANalytical, Almelo, The Netherlands) with CuKα radiation (λ = 1.5406 Å, 40 kV, 15 mA). Data were collected over a 2θ range of 10–60° (step size 0.02°, counting time 1 s/step). Samples were oven-dried to a constant mass and sieved to <63 µm. Phase identification was carried out using HighScore Plus (v. 4.8) and the ICDD PDF-4+ database. The particle size distribution of the powdered samples was analyzed by laser diffraction using an Anton Paar PSA instrument (Anton Paar GmbH, Graz, Austria). Measurements were performed within the particle size range of 0.01–1000 μm. 

**Figure 1:**
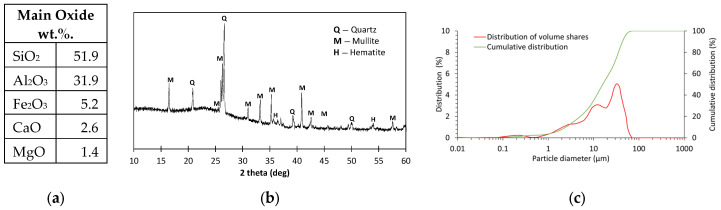
FA: (**a**) main oxide components; (**b**) XRD diffractogram; (**c**) particle size distribution.

The geopolymer mortar used as the matrix for the fibre pull-out test was prepared using the following components. FA with more than 80% silicon and aluminium oxides. Quartz, mullite and haematite are mainly crystalline phases found by XRD analysis. The average diameter of FA particles determined by laser diffraction was 19.9 µm. A detailed FA characterisation including oxide composition, XRD analysis and particle size distribution is shown in Figure 1.

**Figure 2:**
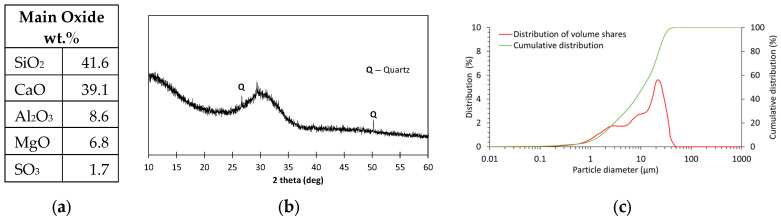
GGBFS: (**a**) main oxide components; (**b**) XRD diffractogram; (**c**) particle size distribution.

A similar analysis was carried out for the GGBFS used. GGBFS is a raw material with an amorphous structure containing more than 40% calcium oxide. Compared to FA, it is characterized by a finer particle size. The average grain diameter of the GGBFS was 13.6 µm. The full characteristics of the GGBFS are shown in Figure 2.

### 2.2. Water Treatment Sludge

**Figure 3:**
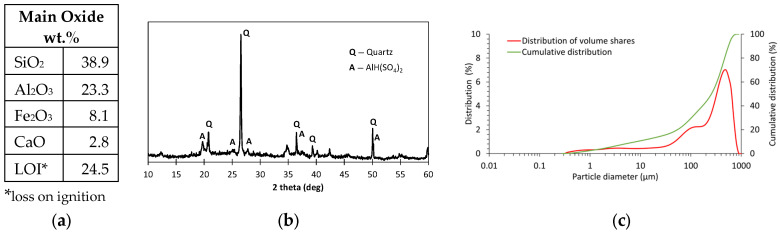
WTS: (**a**) main oxide components; (**b**) XRD diffractogram; (**c**) particle size distribution.

During water treatment processes to produce potable water, large amounts of residue known as water treatment sludge are generated. WTS shows considerable potential as a component of mineral binders. Due to its high content of silicon and aluminum oxides, WTS can be used both as a partial binder substitute and as fine aggregate. The use of WTS contributes to the reduction of natural resource consumption, lowers production costs, and decreases the carbon footprint, while simultaneously enabling the management of a problematic waste material [45]. In this study, sludge from a municipal water treatment plant was used. The sludge was produced during water coagulation with aluminium coagulant (PAX) and sand filtration. The WTS contains mainly silicon, aluminium and iron oxides. The composition of the WTS and the main properties are shown in Figure 3.

**Figure 4:**
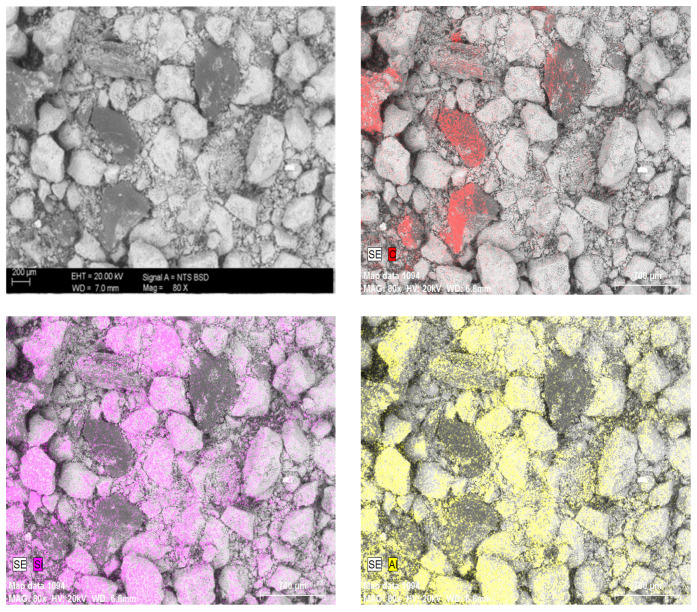
EDS mapping showing the main elements in the WTS grains: C, Si, Al.

The main reason for the high loss of ignition (LOI) is the significant share of activated carbon added during the water treatment processes. In LOI calculation, represents more than 24% of the sludge mass. In order to further verify the composition due to the share of activated carbon in the WTS, analysis was performed using a JEOL IT200 scanning electron microscope (JEOL Ltd., Tokyo, Japan) equipped with an EDS detector. The measurements were conducted on dried and finely ground WTS samples, which were fixed on aluminum stubs using conductive silver paste to avoid carbon contamination from the mounting medium. The analysis provided semi-quantitative information on the elemental composition. The mapping presented in Figure 4 clearly shows the presence of activated carbon grains highlighted in red. The other grains are mainly aluminosilicate minerals. The literature review shows that the use of water treatment sludge, a waste material, as a precursor for geopolymer matrix production has been reported in only a very limited number of publications [46,47,48,49]. The number of studies becomes even more restricted when hydrated WTS is taken into account. It should be emphasized that, due to the generally limited feasibility of calcining this material under industrial conditions, the research described below involved an attempt to use it in its natural form, that is, highly hydrated and not thermally amorphized.

### 2.3. Alkaline Activator

A sodium silicate solution was used as an activator to prepare the geopolymer paste. The ready-to-use product, marketed under the trade name Geosil, is manufactured by Woellner. This solution has an optimized composition and is specifically designed for alkali-activated materials. The technical parameters of the product specified by the manufacturer are given in Table 1.

### 2.4. Steel, PP and Basalt Fibres

The adhesion test results between various types of fibres and geopolymer matrices presented in the article are intended to serve as a starting point for a more informed selection, both in terms of material and geometry (i.e., fibre length and shape) of dispersed reinforcement in GP concretes. The choice of fibres tested was based on their commercial availability and on prior extensive verification of their suitability for use in cement concrete technology. Fibres made from different materials: steel, polypropylene, and basalt and differing in shape, provide a broad perspective on the key issue of their anchorage in geopolymer matrices and, consequently, at a later stage, on the values of residual stresses in reinforced concretes.

MiniBars™ solution is a high-performance composite macrofibre, based on basalt and engineered for concrete reinforcement. Basalt-based MiniBars with a helix shape, length of 55 mm and a diameter of 0.7 mm protected by thermoset resin are manufactured by ReforceTech. The second type of fibre was structural steel fibres for concrete reinforcement. The product called BAUMIX^®^ 60 is a shaped steel fibre with a length of 60 mm and a diameter of 1.0 mm made of cold-drawn low-carbon steel. The third fibre tested was PoliArm PP fibres, 55 mm long and 1.0 mm in diameter. PoliArm is a structural synthetic macrofibre for concrete reinforcement. It is a separate hard fibre with a sinusoidal-wave shape made of oriented polypropylene. The tensile strength of the Minibars’ fibres was determined in the laboratory based on our own measurements. The value used for further calculation was the average of the three results. The tensile strength of the other fibres was taken from the manufacturer’s data. The assumed anchorage length for each fibre has been determined by preliminary tests. The aim was to estimate the length at which it was possible to pull the fibre out of the matrix without breaking it. The fibre parameters are shown in Table 2.

### 2.5. Compositions and Samples of Geopolymer Mortars

The scope of the research included an analysis of fibre adhesion in seven different geopolymer mortars. The mineral precursor of the mixtures consisted of FA and GGBFS. As an activator, the alkaline sodium silicate solution was used. The aggregate was quartz sand with a particle size of up to 2 mm. The first four mixtures differed in the proportions of GGBFS and FA in the precursor composition, ranging from 10% GGBFS and 90% FA to 50% GGBFS and 50% FA by weight. The remaining three mixtures were prepared by gradually replacing FA with WTS. Based on the mechanical strength results, a blend with proportions of 40%GGBFS and 60%FA was selected for modification. WTS was then introduced into the mix precursor composition at 20%, 40% and 60% replacing FA. The detailed compositions of the mixtures are presented in Table 3.

The process of preparing the geopolymer mortar was carried out in stages. In the first stage, an alkaline solution was prepared by adding water to Geosil 34417 in quantities consistent with the mix design assumptions. Next, the solution was added to the FA and GGBFS, and the components were thoroughly mixed. In the following step, WTS was introduced in amounts depending on the specific mix variant. The final component added to the mortar was sand. The total mixing time was 8 min. Setting times were determined using an automatic Vicat apparatus, Vicamatic 3 by Controls. The initial setting time was defined as the moment when the needle reached a distance of 6 mm from the base plate, measured from the start of mixing. Final setting was defined as the point at which the needle penetrated the sample to a depth of only 0.5 mm. Depending on the mix composition, initial setting occurred between 1:30 and 5:00 min, and final setting between 1:50 and 7:30 min. It was observed that a higher GGBFS content accelerated the setting process. The flexural and compressive strengths of the geopolymer matrix were determined using the standard procedure for cement mortars, in accordance with PN-EN 196-1:2016 [50]. For each mortar type, three 40 × 40 × 160 mm beams were prepared and tested 28 days after casting. Flexural strength was first determined using a three-point bending test. Subsequently, the resulting six half-beams were used to determine compressive strength.

### 2.6. Pull-Out Test

**Figure 5:**
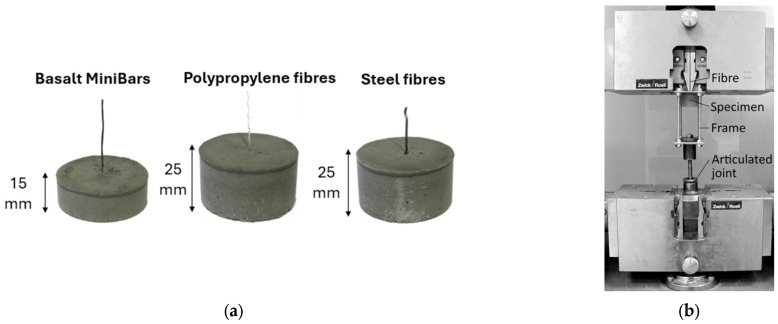
(**a**) Samples; (**b**) adhesion test stand.

Cylindrical moulds with an internal diameter of 50 mm and heights of 15 and 25 mm were made for the pull-out tests of the fibres from the geopolymer matrix. A hole had been made in the bottom of each mould to allow a single fibre to be anchored. This ensured that the fibre kept its axial position during the filling of the mould with the geopolymer mix. Once the required matrix strength was achieved, the fibres were cut from the bottom of the mould, and the base was removed. The protruding end of each fibre was secured between two holders using resin adhesive, which prevented the fibres from slipping out of the plates during testing. Additionally, the plates were positioned adjacent to the surface of the geopolymer matrix in such a way that the recorded elongation resulted solely from the deformation of the fibre within the specimen and its displacement relative to the geopolymer mortar. As previously mentioned, the anchoring of fibres in the tested geopolymer matrices is influenced not only by adhesion phenomena, i.e., the bonds resulting from the surface energy of both the matrix and the fibres. Equally important is their complex shape, which in practice leads to a non-uniform distribution of shear stresses along the interfacial zone between the matrix and the inclusion. This, in turn, necessitates an appropriate selection of the embedding depth of the tested fibres in the matrix, from which they are extracted during pull-out tests. On one hand, too shallow anchoring of the fibre results in high sensitivity of measurements to any imperfections, such as deviations from axial alignment during testing or disturbances in the interfacial zone in the near-surface layers caused by sample preparation. On the other hand, excessive embedding depth of the fibres in the matrix may lead to significant deformations, especially necking or even fibre breakage during measurement, thus preventing the determination of anchoring force. Preliminary tests conducted prior to the main research enabled the selection of the most favourable embedding depth, i.e., 25 mm for steel and PP fibres, and 15 mm for basalt fibres—see Figure 5a.

Three samples were prepared for each fibre type. The moulded samples with fibres were stored under laboratory conditions for 28 days.

The mechanical pull-out test was conducted using a Zwick/Roell Z050 (ZwickRoell GmbH & Co. KG, Ulm, Germany) testing machine, which enables simultaneous recording of both force and fibre elongation. The assumed test velocity was 1 mm/min. The test stand and specimen holding method are shown in Figure 5b.

The recorded curves of the force–displacement relationship allowed the following parameters to be determined:

Maximum force F_max_—directly from the graph

Maximum shear stress τ_max_ based on the relationship:

(1) $$\tau_{max}=\frac{F_{max}}{S}[kPa]$$

where:

F_max_—maximum force [kN]
S—fibre/matrix contact area [m^2^]
W_pull-out_—fibre pulling work based on the relationship:

(2) $$W_{pull-out}=\int_{0}^{\delta_{max}}Fd\delta [mJ]$$

where:

F—force [kN]
δ_max_—maximum elongation—directly from the graph

**Figure 6:**
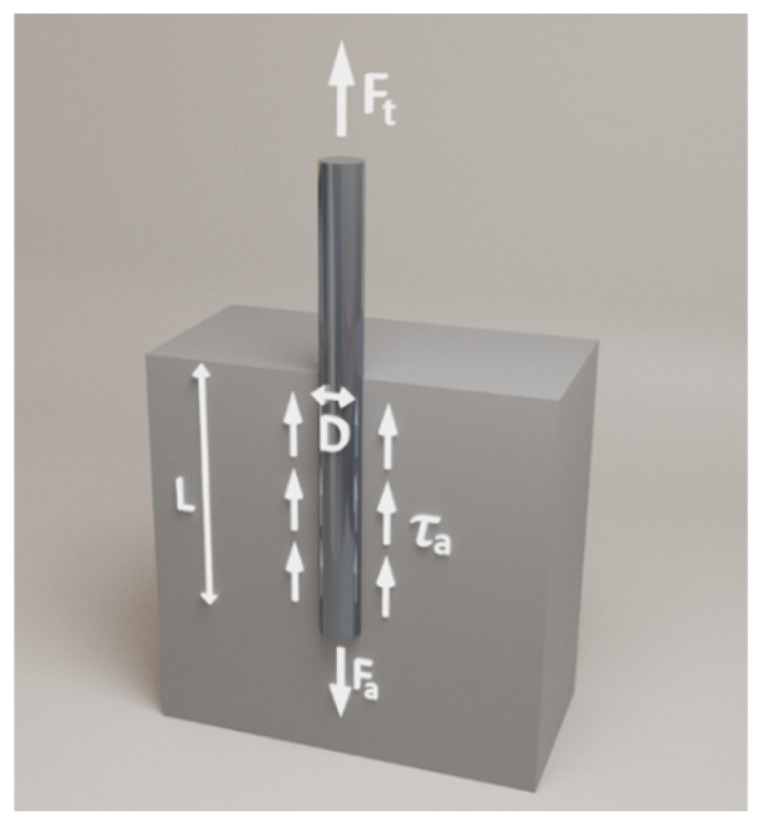
The distribution of forces and stresses in the fibre-reinforced composites.

The distribution of forces and stresses in the specimen during fibre pull-out is shown in Figure 6. For the fibre critical length calculation, the maximum shear stress values were chosen, i.e., calculated with an anchorage of 25 mm or 15 mm, depending on the type of fibre.

Balancing the fibre breaking force and the adhesion force in relation to the matrix

(3) $$F_{t}=F_{a}$$

In the case of a cylindrical fibre:

(4) $$f_{t}\cdot \pi \cdot \frac{D^{2}}{4}=\tau_{a}\cdot \pi \cdot D\cdot L$$

(5) $$L=\frac{f_{t}\cdot D}{4\cdot \tau_{a}}$$

Critical length of a cylindrical fibre:

(6) $$L_{c}=2\cdot L=\frac{f_{t}\cdot D}{2\cdot \tau_{a}}$$

Critical length of any fibre shape:

(7) $$L_{c}=\frac{{2\cdot A\cdot f}_{t}}{\tau_{a}\cdot P_{f}}$$

where:

A—fibre cross-sectional area;
P_f_—fibre perimeter
f_t_—fibre tensile strength
τ_a_—tangential stress (adhesion)
D—Fiber diameter

## 3. Results and Discussion

The flexural and compressive strength results highlight the significant role of GGBFS in the development of composite strength. The addition of GGBFS to the geopolymer significantly influences the microstructure and the development of phases such as N-A-S-H (Sodium Aluminium Silicate Hydrate) and C-A-S-H (Calcium Aluminium Silicate Hydrate) gels. The introduction of calcium-rich GGBFS enhances the formation of C-A-S-H phases, which occur together with the N-A-S-H gels typical of FA-based geopolymers. The presence of both gels results in a matrix with increased density and reduced porosity, leading to improved mechanical properties and enhanced chemical resistance. Additionally, the high reactivity of GGBFS in an alkaline environment accelerates the setting and curing processes, while also contributing to long-term structural stability [18,51]. 

**Figure 7:**
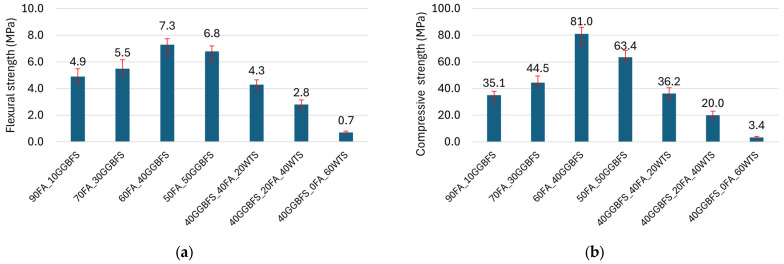
Geopolymer mortars: (**a**) flexural strength and (**b**) compressive strength.

As shown in Figure 7, an increase in GGBFS content in the mixture led to improved strength, up to an optimal level of 40% GGBFS. At this content, the compressive strength exceeded 80 MPa, while the flexural strength was greater than 7 MPa. Compared to the composite containing 10% GGBFS, the observed compressive strength values were more than twice as high, while the flexural strength increased by approximately 50%. A further increase in the amount of GGBFS results in a reduction of the determined mechanical properties. For this reason, blends incorporating WTS were developed based on the most advantageous composition, i.e., 60FA_40GGBFS. Replacing FA with WTS resulted in a rapid decrease in strength. The introduction of a 20% WTS precursor into the composition resulted in a decrease in compressive strength of more than 50%. When FA was completely replaced by WTS, the compressive strength was less than 5% of the reference value (i.e., the mixture without WTS). The study shows that despite the high content of SiO_2_ and Al_2_O_3_ in the WTS composition, above 60%, the strongly crystallized form of silica limits its reactivity in the alkaline environment of the geopolymer matrix. The XRD results shown in Figure 3b highlight intensive peaks indicative of the presence of inert β-quartz. The results of the mechanical properties of the mortars confirm that the use of non-calcined waste WTS as a precursor for geopolymer matrix formation is an unfavourable solution.

### 3.1. Basalt Fibres

**Figure 8:**
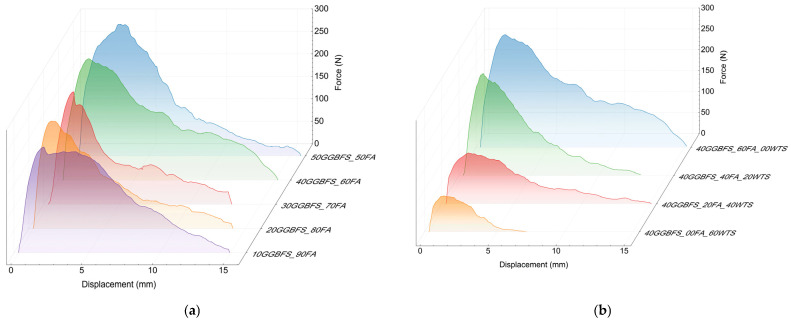
Results of the Minibars pull-out test: (**a**) matrix with different ratios between GGBFS and FA, (**b**) matrix with different WTS contents.

The basalt Minibars showed the lowest deformability before the maximum shear stress was reached. In other words, the angle of inclination up to the proportional limit with regard to the displacement axis was the highest. However, this does not mean that the basalt fibre has the highest modulus of elasticity relative to the other fibres tested, but rather this phenomenon can be attributed to the straight shape of the fibre. As a consequence, a uniform shear stress distribution is observed along the entire length of the fibre immersed in the tested composite. In the case of other fibres, more complex shapes result in greater matrix deformation and, consequently, increased deformability during the stage of rising recorded force. The simple shape of the basalt fibres is also the reason for the stable and almost linear decrease in post-critical stresses, i.e., those recorded after decohesion of the inclusion into the matrix. The reduction in recorded force can be attributed to the progressive loss of anchorage, and thus friction, between the fibre and the matrix. The results are summarised in Figure 8.

Analysis of the obtained forces and calculated adhesion reveals a clear relationship with the composition of the geopolymer matrix (see Table 4). As the GGBFS content in the mixture increases, adhesion improves. The highest adhesion value of 8.9 MPa was observed for a mix whose precursor contained the maximum content, i.e., 50% of GGBFS. On the other hand, the addition of WTS clearly reduces adhesion. For the mixture in which WTS replaced 40% of FA in the precursor composition (40GGBFS_20FA_40WTS), adhesion decreased by 65% compared to the reference value (60FA_40GGBFS). The complete replacement of the FA by the WTS has resulted in an even greater decline. For each mixture, critical fibre lengths were calculated based on the fibre diameter, tensile strength, and maximum recorded tangential stress value. For the geopolymer matrix that exhibited the best interaction with the fibre (50FA_50GGBFS), the critical length was approximately 57 mm.

**Figure 9:**
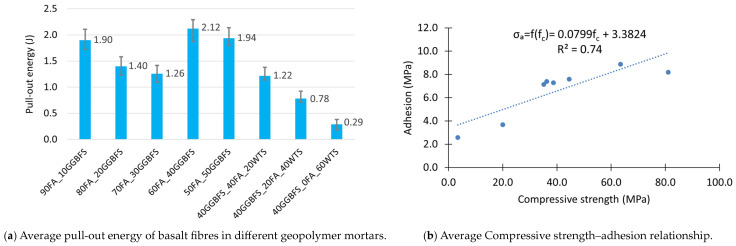
Adhesion test results: (**a**) pull-out energy; (**b**) compressive strength–adhesion correlation.

When analyzing the pull-out energy parameter, it is important to remember that the total amount of energy required to remove a fibre involves both pulling the fibre out and stretching it inside the matrix. Also in this case, the mixtures containing the largest amounts of GGBFS (60FA_40GGBFS and 50FA_50GGBFS) required the most energy to pull all the fibre out of the matrix (Figure 9a). 

It is also worth pointing out that local deformation occurs during the test, as well as the formation of many micro-cracks and consequently, the partial destruction of the geopolymer matrix. Hence, the energy required to pull out the fibres should be correlated to some extent with the strength of the composite. Figure 9b shows the relationship between fibre adhesion and matrix compressive strength. The presented graph confirms a quite good fit of the linear model describing this relationship (the value of the determination coefficient R^2^ is 0.74). As can be seen from the equation presented in Figure 9b, an increase in the compressive strength of the geopolymer matrix by 10 MPa results in an estimated increase in adhesion of 0.8 MPa.

### 3.2. Steel Fibres

**Figure 10:**
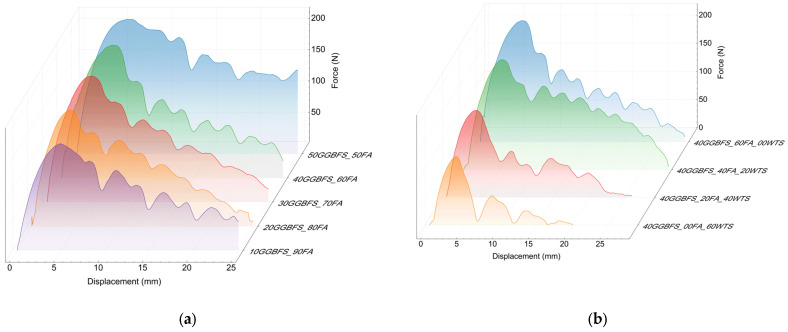
Results of the steel fibre pull-out test: (**a**) matrix with different ratios between GGBFS and FA and (**b**) matrix with different WTS contents.

In the case of steel fibres with bent ends, a relatively high level of maximum force values was observed (Figure 10). This indicates good interaction with the matrix during fibre pull-out. The observed irregular curve in the post-critical stress region is manifested by alternating decreases and increases in force. This phenomenon is related not only to the displacement of the hooked end of the fibre, which causes local matrix failure and subsequent re-anchoring at random locations, but also to the progressive straightening of the fibre. Thus, the post-maximum shear stress interaction between the steel fibre and the matrix is primarily attributed to the fibre’s shape.

Among all the fibres tested, steel fibres exhibited the highest adhesion to the geopolymer matrix. For the 50FA_50GGBFS matrix, the value was 9.3 MPa, and the calculated critical length at this stress was approximately 55mm (see Table 5). Analogously to basalt fibres, the effect of the mix composition on fibre adhesion is clearly visible. With increasing GGBFS content, the adhesion improves. In contrast, the addition of WTS similarly decreases adhesion. The low matrix strength of a few MPa entails the need to extend the anchorage of both the steel and basalt fibres, i.e., to increase their critical length to as much as about 200 mm. Due to the entanglement effect of fibres during the production of the reinforced composite mix, the use of longer fibres is precluded. Maintaining a relatively straight shape of the fibre in the final concrete mix depends on its stiffness; however, the fibre length typically does not exceed approximately 60 mm.

**Figure 11:**
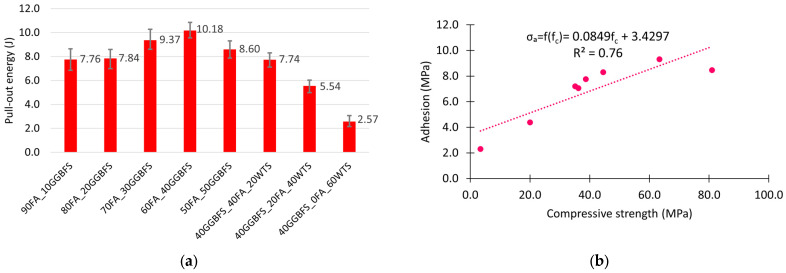
Adhesion test results: (**a**) pull-out energy; (**b**) compressive strength–adhesion correlation.

The good interaction between the steel fibre and the geopolymer matrix is confirmed by the high energy required to pull the fibre out of the matrix. Figure 11a shows a bar chart of this energy for the different matrices. As with basalt fibres, their adhesion to the matrix is not only dependent on the bonds between the phases, but also, due to their hooked end, on the strength of the matrix. Figure 11b shows the relationship between steel fibre adhesion on the matrix compressive strength. The linear model used describes this relationship slightly better than for basalt fibres. The value of the determination coefficient R^2^ is 0.76. An increase in geopolymer matrix strength of 10 MPa for steel fibres results in an increase in adhesion of 0.85 MPa.

### 3.3. Polypropylene Fibres

**Figure 12:**
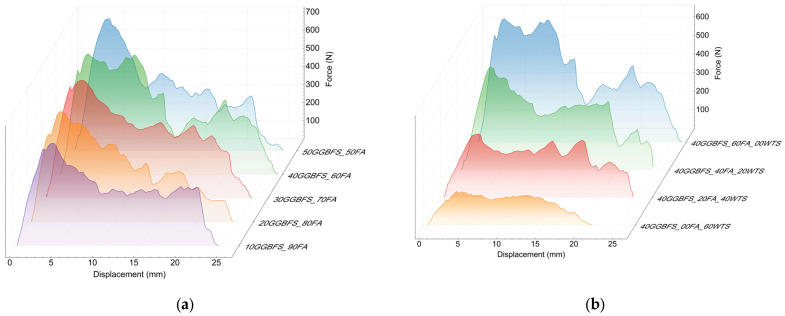
Results of the PP fibres pull-out test: (**a**) matrix with different ratios between GGBFS and FA and (**b**) matrix with different WTS contents.

PP fibres showed the lowest F_max_ values of all the fibres tested. The highest adhesion among all the fibres tested was observed in the matrix in which 50% FA and 50% GGBFS were used as precursors. However, although the difference between basalt and steel fibres in this case was not significant, amounting to only about 5%, the adhesion of PP fibres was much lower, at 2.8 MPa, which corresponds to only 30% of the value achieved by steel fibres. Nevertheless, the relatively low tensile strength of the PP fibre, also about 35% of the steel fibre, meant that the determined critical length of the PP fibre is not significantly greater compared to steel or basalt. In the 50FA_50GGBFS matrix for steel, L_c_ = 55m, while for PP fibre, L_c_ only extended to 65 mm. The force–displacement curves shown in Figure 12 in the post-critical area clearly show a cyclical character. This is reflected in the shape of the fibres and, in fact, in the manner in which they interact with the geopolymer matrix. The observed fibre pull-out mechanism involved the simultaneous straightening of the fibre crimp and the formation of cracks in the surrounding matrix, which resulted in a decrease in the recorded force. Subsequently, before a similar destruction of the cooperating fibre with the matrix occurred in the lower layer of the tested sample, a local increase in the force necessary to initiate the next cycle described above was recorded. Both the number of cycles and their regularity decreased with the decrease in the strength of the matrix. Probably the area of damage during the straightening of the next PP fibre bend in the matrix was significantly larger.

The adhesion of PP fibres was by far the lowest. In this case, the composition of the geopolymer mix did not have a significant effect on the adhesion values. Excluding the mix with the highest WTS content (40GGBFS_0FA_60WTS), the calculated adhesion values were between 2.0 and 2.8 MPa. This also results in very similar critical fibre lengths, with a value in the range between about 65 and 90 mm (Table 6).

**Figure 13:**
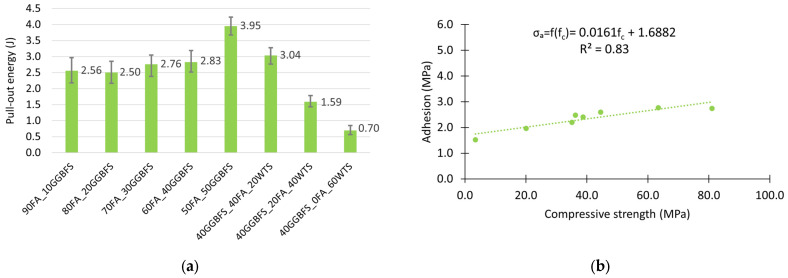
Adhesion test results: (**a**) pull-out energy and (**b**) compressive strength–adhesion correlation.

The calculated pull-out energy values are approximately three times lower than those obtained for steel fibres. They are, however, greater than the results obtained for basalt Minibars fibres. Nevertheless, it should be noted that the anchorage length of the minibars was reduced due to their linear shape. The higher result may also be attributed to the wavy shape of the PP fibres, which generates greater resistance during fibre pull-out, as described above, thereby increasing the amount of energy required for this process. The results are shown in Figure 13a. Given the way in which the wavy PP fibre interacts with the matrix, i.e., its cyclic crushing in the surroundings of a single bend, the strength of the matrix will play a particularly significant role in this case. It seems reasonable that the value of the determination coefficient in the presented model of the linear relationship between adhesion and compressive strength of the matrix has the highest value of R^2^ = 0.83. The equation presented in Figure 13b confirms that the investigated range of geopolymer matrix strength, an average increase of 10 MPa in compressive strength, involves an increase of 0.16 MPa in adhesion.

### 3.4. SEM Observation

**Figure 14:**
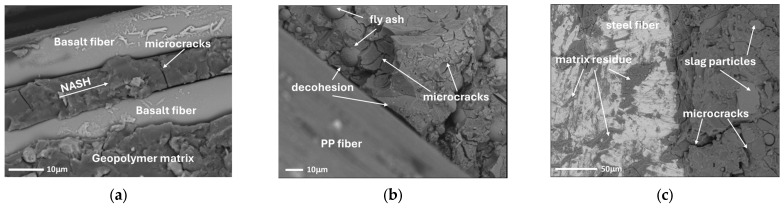
SEM image analysis: (**a**) basalt fibre; (**b**) PP fibre; (**c**) steel fibre.

To illustrate the differences in bonding between the various types of fibres and the geopolymer matrix, SEM observations were conducted. The fibre-reinforced geopolymer specimens were fractured to expose the fibre–matrix interface, allowing direct observation of the interfacial region. The samples were analyzed in their natural state, without additional preparation. Observations were carried out in high-vacuum mode at an accelerating voltage of 15–20 kV, using SE and BSE detectors. Figure 14 shows the interfacial transition zone between basalt, PP, steel fibres and the geopolymer matrix. It can be observed that there are differences in the matrix–fibre contact zone for each type of fibre, which explains the calculated adhesion values.

The scanning microscope images show the structure of the geopolymer composite with basalt fibres (Figure 14a). No traces of hydrated aluminosilicates were observed on the fibre surfaces, indicating limited interaction with the matrix. A clear decohesion can be seen at the fibre–matrix interface. The surface of the fibres remains smooth after destruction, confirming their poor adhesion to the matrix. This is due to the composite fibre structure, where basalt microfibres are embedded in resin. A homogeneous, amorphous N-A-S-H phase is clearly visible within the geopolymer structure. This phase is characteristic of the products formed during the alkaline activation of aluminosilicate raw materials. In addition, single microcracks can be observed in the matrix in the area of the fibres. Their presence suggests the occurrence of shrinkage deformations, which may result from the intrinsic properties of the geopolymer or from the stress transfer mechanisms within the composite.

The SEM image in Figure 14b shows the structure of the geopolymer composite reinforced with PP fibres. No traces of paste were observed on the fibre surfaces, suggesting a lack of chemical interaction between the fibres and the geopolymer matrix. A pronounced decohesion at the fibre–matrix interface was observed, indicating low adhesion of the PP fibres to the geopolymer matrix and a limited ability to transfer stresses through adhesion. Numerous shrinkage microcracks are visible in the geopolymer matrix, which may result from stresses generated during the setting and hardening processes. In addition, the presence of amorphous spherical silica particles from the FA was observed. They are typical residues from the alkaline activation reaction. The occurrence of unreacted precursor particles, specifically FA grains, can be attributed to the limited setting time of the mixture. The incorporation of a blended precursor system comprising FA and GGBFS enables the synthesis of a binding composite under ambient conditions, eliminating the need for additional thermal curing. However, this modification substantially reduces the setting time, which in turn leads to incomplete reaction of the FA component. As a result, the full reactive potential of the raw material remains unutilized.

The last microscopic image (Figure 14c) shows the structure of the steel fibre reinforced geopolymer composite. On the left, the steel fibre is visible with many traces of the geopolymer matrix attached to its surface, indicating good adhesion between the fibre and the geopolymer paste. The fibre–matrix contact zone is homogeneous and free of cracks and voids, showing the excellent adhesion of the geopolymer to the steel, contributing to effective stress transfer in the composite. The structure of the geopolymer matrix is consistent with observations reported for other cases—numerous shrinkage microcracks are visible, resulting from the physical and chemical processes taking place. In addition, relics of irregularly shaped GGBFS particles and spherical grains of amorphous microsilica from FA were observed in the matrix. Microstructural analysis revealed partially dissolved GGBFS relics within the geopolymer matrix. While raw GGBFS particles typically exhibit sharp-edged morphology, the observed grains displayed smoothed or rounded edges, indicating partial dissolution in the alkaline environment. The presence of unreacted precursor particles is likely associated with several processing parameters, including the aforementioned short setting time, as well as low activator concentration—formulated without sodium hydroxide—and ambient-temperature curing without additional heating, all of which may have limited precursor dissolution.

## 4. Summary and Conclusions

Replacing FA with WTS resulted in a significant reduction in strength in the tested composites. Incorporating 20% WTS into the mix led to a compressive strength reduction of over 50%. When FA was fully replaced by WTS, the compressive strength dropped to less than 5% of the reference value (the strength of the equivalent mortar without WTS). This decrease in strength can be attributed to the differing properties of the two raw materials. WTS, as a mineral precursor under alkaline activation, exhibits lower reactivity. Its high content of crystalline phases, mainly quartz, larger grain size, and significant loss on ignition (approximately 25%) contribute to its low potential for alkaline activation. It should be emphasized that the obtained test results and the drawn conclusions refer exclusively to geopolymer composites produced using only a sodium silicate solution as the activator and cured under ambient conditions. Authors of publications [52,53] show that different outcomes can be expected for composites prepared with hydroxides as an activator and cured at elevated temperatures. In summary, the results of the mortar strength tests indicate that non-calcined WTS acts more as a mineral filler in the discussed mixtures rather than as a precursor involved in the formation of geopolymerisation products. However, its chemical affinity toward alkaline ions still requires further complementary investigation.

**Figure 15:**
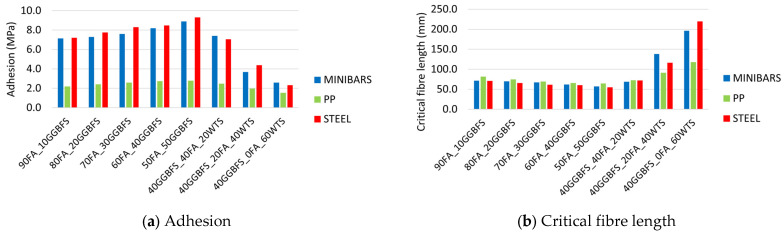
Summary of the pull-out test.

Figure 15 summarizes all types of fibre adhesion and calculated critical fibre lengths obtained for each geopolymer matrix. Based on the results, trends describing the influence of geopolymer composition and fibre type on matrix–fibre interactions can be identified. The analyzed basalt, steel, and PP fibres exhibit qualitatively distinct behaviours in contact with the geopolymer matrix. When pulling out the PP fibres, a kind of cyclicity in the force–displacement relationship was observed due to the straightening of the crimp in the geopolymer matrix. For steel fibres, a slightly different effect was associated with the displacement of the bent ends, manifested by a more irregular, broken curve in the post-critical region of the force–displacement relationship. In both cases, the shape of the fibres was an important determinant of the fibre–matrix interaction. The basalt Minibars, which are helix-shaped, showed a rapid decrease in recorded force after reaching their maximum value, which can be explained by the decrease in friction between the fibre and the geopolymer with decreasing depth of embedment. 

The highest values of force, and consequently shear stress, were observed for steel fibres. Steel fibres also exhibited the highest energy required for fibre pull-out, primarily due to their shape and the excellent adhesion of steel to the geopolymer matrix. In mixtures without the addition of WTS, the calculated critical lengths for all analyzed fibres indicate that steel fibres have the lowest value. This suggests that steel fibres possess the most favourable tensile strength-to-adhesion ratio with respect to the tested geopolymer matrix. The analysis conducted for the weakest matrices containing WTS revealed the lowest critical lengths for PP fibres. In the mechanically weakest matrix, the crimped shape of the PP fibres had a dominant influence on their interaction with the geopolymer mortar. In the case of matrices containing WTS, the mechanical properties of the geopolymer are significantly lower. This consequently leads to a reduction in the adhesion of the dispersed reinforcement and thus an increase in their critical length.

The completed pull-out tests performed for three types of fibre, basalt, steel, and PP, anchored at depths of 15 or 25 mm in different geopolymer matrices, allowed the following conclusions to be drawn:

- The presence of WTS as a precursor in the geopolymer binder of mortars significantly reduces their mechanical properties. An increase of this waste material content by 10% causes a decrease in flexural strength of approximately 1 MPa, whereas compressive strength is reduced by more than 12 MPa.
- Within the investigated range (10–50%), increasing the GGBFS content in the geopolymer binder increases fibre–matrix adhesion regardless of fibre type. This effect is more pronounced for steel and basalt fibres, where σ_a_ increases on average by approximately 0.5 MPa per 10% rise in GGBFS content, whereas for PP fibres, the increase is only about 0.15 MPa.
- Steel fibres exhibited the greatest sensitivity of adhesion to changes in matrix mechanical properties. Linear regression analysis within the compressive strength range up to 80 MPa showed that an increase of 1 MPa in compressive strength corresponds to an average adhesion increase of 85 kPa. By contrast, PP fibres showed the smallest changes, where the average adhesion increase observed was only 16 kPa.
- The presence of WTS reduces the adhesion of all types of fibres to the geopolymer matrix. The effect is strongest for steel and basalt fibres, where a 10% increase in WTS content in the matrix causes a reduction in adhesion of more than 1 MPa in both cases. For PP fibres, the decrease is approximately 0.2 MPa.
- Steel and basalt fibres exhibit the highest adhesion to the geopolymer matrix free of water treatment sludge, with values around 8–9 MPa. PP fibres show substantially lower adhesion, approximately one-third of that of steel and basalt, at below 3 MPa.
- Too-short fibres used to reinforce brittle mineral composites can increase flexural strength but typically provide low toughness. Conversely, overly long fibres tend to tangle, which can reduce flexural strength while often enhancing composite ductility. The results obtained allow for the determination of the optimum length for the studied fibres in geopolymer matrices. The critical length for basalt, steel, and PP fibres in matrices without WTS is approximately 50–70 mm. These values indicate that commercially produced fibres with lengths close to the reported range can be effectively used to manufacture reinforced mortars and concretes based on geopolymer matrices.
- SEM microscopic observations showed the presence of unreacted precursor particles, microcracks in the geopolymer matrix. In addition, particularly good adhesion of steel fibres is confirmed by the presence of matrix traces on the fibre surface and the integrity of the matrix–fibre contact zone.

In conclusion, incorporating steel and basalt fibres into geopolymer mortars and concretes offers a promising approach to mitigating brittleness by enhancing the material’s microstructure, limiting crack development, and improving ductility and structural performance. Fibre-reinforced geopolymer composites hold significant potential for applications in infrastructure and construction. Nevertheless, before such solutions can be widely implemented, further studies are necessary to verify the feasibility of uniformly distributing dispersed reinforcement within geopolymer mixtures and to assess the extent of composite enhancement, such as through tensile strength at bending tests, along with monitoring of residual stress.

## Figures and Tables

**Table 1:** Woellner Geosil 34417. Data provided by the manufacturer.

Characteristic	Woellner Geosil 34417
Na_2_O content (wt.%)	16.74
SiO_2_ content (wt.%)	27.5
Density (g/cm^3^)	1.552
Viscosity (mPa·s)	470
Weight ratio (WR = wt.% SiO_2_/wt.% Na_2_O)	1.64
Molar ratio (MR = mol SiO_2_/molNa_2_O)	1.70

**Table 2:** Properties of the fibres used.

Fibres	Anchorage Length [mm]	Diameter [mm]	Perimeter [mm]	Fibre–Matrix Contact Surface [mm^2^]	Tensile Strength [MPa]	Shape
**MiniBars**	15	0.7	2.2	33.0	1456	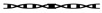
**PP**	25	1.0	3.1	77.5	360	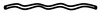
**Steel**	25	1.0	3.1	77.5	1020	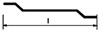

**Table 3:** Geopolymer mortar compositions in grams per litre.

Component [g]	10GGBFS/90FA	30GGBFS/70FA	40GGBFS/60FA	50GGBFS/50FA	40GGBFS/40FA/ 20WTS	40GGBFS/20FA/ 40WTS	40GGBFS/0FA/ 60WTS
Alkaline solution	250	250	250	250	250	250	250
Water	120	120	120	120	0	0	0
FA	720	560	480	400	320	160	0
GGBFS	80	240	320	400	320	320	320
WTS (dry material)	0	0	0	0	160	320	480
Sand 0/2 mm	1200	1200	1200	1200	1200	1200	1200

**Table 4:** Basalt fibres adhesion test results.

Geopolymer Mix	Maximum Force F_max_		Adhesion σ_a_ (MPa)	Critical Fibre Length L_c_ (mm)
	(N)	Coefficient of Variation V (%)		
90FA_10GGBFS	235.7	4.8	7.1	71.3
80FA_20GGBFS	241.6	4.0	7.3	69.8
70FA_30GGBFS	250.8	3.9	7.6	67.0
60FA_40GGBFS	270.7	4.9	8.2	62.1
50FA_50GGBFS	293.3	5.3	8.9	57.3
40GGBFS_40FA_20WTS	244.3	4.4	7.4	68.8
40GGBFS_20FA_40WTS	121.8	4.6	3.7	138.0
40GGBFS_0FA_60WTS	85.6	6.6	2.6	196.3

**Table 5:** Steel fibres adhesion test results.

Geopolymer Mix	Maximum Force F_max_		Adhesion σ_a_ (MPa)	Critical Fibre Length L_c_ (mm)
	(N)	Coefficient of Variation V (%)		
90FA_10GGBFS	565.5	5.8	7.2	70.8
80FA_20GGBFS	609.5	6.3	7.8	65.7
70FA_30GGBFS	651.7	5.6	8.3	61.4
60FA_40GGBFS	664.9	5.2	8.5	60.2
50FA_50GGBFS	730.7	4.8	9.3	54.8
40GGBFS_40FA_20WTS	554.4	6.0	7.1	72.2
40GGBFS_20FA_40WTS	343.8	6.3	4.4	116.4
40GGBFS_0FA_60WTS	182.0	6.1	2.3	219.9

**Table 6:** PP fibres adhesion test results.

Geopolymer Mix	Maximum Force F_max_		Adhesion σ_a_ (MPa)	Critical Fibre Length L_c_ (mm)
	(N)	Coefficient of Variation V (%)		
90FA_10GGBFS	173.1	5.5	2.2	81.7
80FA_20GGBFS	189.0	5.7	2.4	74.8
70FA_30GGBFS	204.0	6.1	2.6	69.3
60FA_40GGBFS	215.2	7.4	2.7	65.7
50FA_50GGBFS	217.9	5.8	2.8	64.9
40GGBFS_40FA_20WTS	195.2	8.1	2.5	72.4
40GGBFS_20FA_40WTS	154.7	8.0	2.0	91.4
40GGBFS_0FA_60WTS	119.8	8.3	1.5	118.0

## Data Availability

The original contributions presented in this study are included in the article. Further inquiries can be directed to the corresponding author.
